# Diagnosis of Non-Celiac Gluten Sensitivity (NCGS): The Salerno Experts’ Criteria

**DOI:** 10.3390/nu7064966

**Published:** 2015-06-18

**Authors:** Carlo Catassi, Luca Elli, Bruno Bonaz, Gerd Bouma, Antonio Carroccio, Gemma Castillejo, Christophe Cellier, Fernanda Cristofori, Laura de Magistris, Jernej Dolinsek, Walburga Dieterich, Ruggiero Francavilla, Marios Hadjivassiliou, Wolfgang Holtmeier, Ute Körner, Dan A. Leffler, Knut E. A. Lundin, Giuseppe Mazzarella, Chris J. Mulder, Nicoletta Pellegrini, Kamran Rostami, David Sanders, Gry Irene Skodje, Detlef Schuppan, Reiner Ullrich, Umberto Volta, Marianne Williams, Victor F. Zevallos, Yurdagül Zopf, Alessio Fasano

**Affiliations:** 1 Department of Pediatrics, Università Politecnica delle Marche, 60123 Ancona, Italy; 2 Centre for the Prevention and Diagnosis of Celiac Disease/Gastroenterology and Endoscopy Unit, Fondazione IRCCS Cà Granda-Ospedale Maggiore Policlinico, Milan 20122, Italy; E-Mail: lucelli@yahoo.com; 3 Clinique Universitaire d’Hépato-Gastroenterologie, CHU de Grenoble, 38043 Grenoble Cedex 09, France; E-Mail: BBonaz@chu-grenoble.fr; 4 Department of Gastroenterology, VU University Medical Center, Amsterdam, the Netherlands; E-Mails: g.bouma@vumc.nl (G.B.); cjmulder@vumc.nl (C.J.M.); 5 Department of Internal Medicine, “Giovanni Paolo II” Hospital, Sciacca (AG) and University of Palermo, Sciacca 92019, Italy; E-Mail: acarroccio@hotmail.com; 6 Paediatric Gastroenterology Unit, Hospital Sant Joan de Reus, 43201 Reus, Spain; E-Mail: gcastillejo@grupsagessa.com; 7 Service d’Hépato-gastro-entérologie et Endoscopie Digestive, Hôpital Européen Georges Pompidou, 75015 Paris, France; E-Mail: christophe.cellier@egp.aphp.fr; 8 Interdisciplinary Department of Medicine, University of Bari, Bari 70124, Italy; E-Mails: fernandacristofori@gmail.com (F.C.); rfrancavilla@gmail.com (R.F.); 9 Department of Internal and Experimental Medicine Magrassi-Lanzara, Second University of Naples, 80131 Naples, Italy; E-Mail: laura.demagistris@unina2.it; 10 Gastroenterology Unit, Department of Pediatrics, University Medical Centre Maribor, Maribor 2000, Slovenia; E-Mail: jernej.dolinsek@ukc-mb.si; 11 Medical Clinic 1, University of Erlangen, 91054 Erlangen, Germany; E-Mails: walburga.dieterich@uk-erlangen.de (W.D.); yurdaguel.zopf@uk-erlangen.de (Y.Z.); 12 Academic Department of Neurosciences and University of Sheffield, Royal Hallamshire Hospital, Sheffield S10 2JF, UK; E-Mail: marios.hadjivassiliou@sth.nhs.uk; 13 Division of Gastroenterology and Internal Medicine, Hospital Porz am Rhein, Köln 51149, Germany; E-Mail: w.holtmeier@khporz.de; 14 Practice of Nutrition Therapy Allergology and Gastroenterology, Köln 50935, Germany; E-Mail: ute.koerner@t-online.de; 15 Division of Gastroenterology, Beth Israel Deaconess Medical Center, Boston, MA 02215, USA; E-Mail: dleffler@bidmc.harvard.edu; 16 Seksjon for Gastromedisin, Avdeling for Transplantasjonsmedisin, OUS Rikshospitalet Senter for Immunregulering, Oslo University, 0424 Oslo, Norway; E-Mail: knut.lundin@medisin.uio.no; 17 Institute of Food Sciences-CNR, Lab. Immuno-Morphology, 83100 Avellino, Italy; E-Mail: gmazzarella@isa.cnr.it; 18 Department of Food Science, University of Parma, IT-43124 Parma, Italy; E-Mail: nicoletta.pellegrini@unipr.it; 19 Department of Gastroenterology, Alexandra Hospital, Redditch B98 7UB, UK; E-Mail: krostami@hotmail.com; 20 Department of Gastroenterology and Hepatology, Royal Hallamshire Hospital and University of Sheffield Medical School, Sheffield S10 2JF, UK; E-Mail: david.sanders@sth.nhs.uk; 21 Division of Clinical Nutrition, Oslo University Hospital, 0424 Oslo, Norway, E-Mail: g.i.skodje@medisin.uio.no; 22 University Medical Center of the Johannes Gutenberg University, 55131 Mainz, Germany; E-Mails: detlef.schuppan@unimedizin-mainz.de (D.S.); zevallos@uni-mainz.de (V.F.Z.); 23 Charité—Universitätsmedizin Berlin, Medizinische Klinik für Gastroenterologie, Infektiologie und Rheumatologie, 12203 Berlin, Germany; E-Mail: reiner.ullrich@charite.de; 24 Department of Medical and Surgical Sciences University of Bologna, St. Orsola-Malpighi Hospital via Massarenti 9, 40138 Bologna, Italy; E-Mail: umberto.volta@aosp.bo.it; 25 Somerset Partnership NHS Foundation Trust, Bridgwater TA6 4RN, UK; E-Mail: marianne@wisediet.co.uk; 26 Pediatric Gastroenterology and Nutrition, Mass General Hospital for Children, Boston, MA 02114, USA; E-Mail: afasano@mgh.harvard.edu

**Keywords:** non-celiac gluten sensitivity, diagnosis, double-blind placebo-controlled challenge, gastrointestinal symptom rating scale, irritable bowel syndrome

## Abstract

Non-Celiac Gluten Sensitivity (NCGS) is a syndrome characterized by intestinal and extra-intestinal symptoms related to the ingestion of gluten-containing food, in subjects that are not affected by either celiac disease or wheat allergy. Given the lack of a NCGS biomarker, there is the need for standardizing the procedure leading to the diagnosis confirmation. In this paper we report experts’ recommendations on how the diagnostic protocol should be performed for the confirmation of NCGS. A full diagnostic procedure should assess the clinical response to the gluten-free diet (GFD) and measure the effect of a gluten challenge after a period of treatment with the GFD. The clinical evaluation is performed using a self-administered instrument incorporating a modified version of the Gastrointestinal Symptom Rating Scale. The patient identifies one to three main symptoms that are quantitatively assessed using a Numerical Rating Scale with a score ranging from 1 to 10. The double-blind placebo-controlled gluten challenge (8 g/day) includes a one-week challenge followed by a one-week washout of strict GFD and by the crossover to the second one-week challenge. The vehicle should contain cooked, homogeneously distributed gluten. At least a variation of 30% of one to three main symptoms between the gluten and the placebo challenge should be detected to discriminate a positive from a negative result. The guidelines provided in this paper will help the clinician to reach a firm and positive diagnosis of NCGS and facilitate the comparisons of different studies, if adopted internationally.

## 1. Introduction

Non-Celiac Gluten Sensitivity (NCGS) is a syndrome characterized by intestinal and extra-intestinal symptoms related to the ingestion of gluten-containing food, in subjects that are not affected by either celiac disease (CD) or wheat allergy (WA) [1,2]. The terminology “NCGS” is still a matter of debate. Although NCGS is triggered by gluten-containing cereals, the offending dietary protein has not been identified yet, and could include component/s that are different from gluten itself, e.g., the cereal protein amylase-trypsin inhibitors (ATIs) [3]. Then the terminology “NCGS” could be changed into “Non Celiac Wheat Sensitivity” (NCWS) in the near future, although this would exclude other relevant cereals like barley and rye. The prevalence of NCGS is not clearly defined yet. Indirect evidence suggests that NCGS is more common than CD [4], the latter affecting around 1% of the general population [5]. Treatment of NCGS is based on the celiac-type gluten-free diet (GFD) although it is unknown if long-term, strict avoidance of all gluten-related products is necessary. Since NCGS may be transient, gluten tolerance needs to be re-assessed in patients with NCGS [6].

Clinical presentation of NCGS might be multi-systemic and there have been a range of signs and symptoms reported in association with this condition (Table 1) [7]. The latency between gluten ingestion and the appearance of symptoms is usually short, within hours or days. Common features of NCGS are symptoms usually diagnosed under the umbrella of the irritable bowel syndrome (IBS), e.g., bloating, abdominal pain and irregular bowel movements [4]. Recent clinical studies have opened new insight into the etiology of these symptoms and the current literature suggests that many of the patients previously known under IBS are in fact intolerant to something they eat. Most common food reactions have been reported to gluten, lactose, milk protein and Fermentable Oligo, Di, and Monosaccharides And Polyols (FODMAPs) [8,9]. NCGS patients, however, often report symptoms outside of the intestinal tract, e.g., headache and/or foggy mind [4], which cannot be accounted for by lactose, and/or FODMAPs intolerance.

**Table 1:** The clinical manifestations of Non-Celiac Gluten Sensitivity (NCGS).

Frequency	Intestinal	Extra-Intestinal
Very Common	Bloating	Lack of wellbeing
	Abdominal pain	Tiredness
Common	Diarrhea	Headache
	Epigastric pain	Anxiety
	Nausea	Foggy mind
	Aerophagia	Numbness
	GER	Joint/muscle pain
	Aphthous stomatitis	Skin rash/dermatitis
	Alternating bowel habits	
	Constipation	
Undetermined	Hematochezia	Weight loss
	Anal fissures	Anemia
		Loss of balance
		Depression
		Rhinitis/asthma
		Weight increase
		Interstitial cystitis
		Ingrown hairs
		Oligo or polymenorrhea
		Sensory symptoms
		Disturbed sleep pattern
		Hallucinations
		Mood swings
		Autism
		Schizophrenia

In recent years, several studies explored the relationship between the ingestion of gluten-containing food and the appearance of neurological and psychiatric disorders/symptoms like ataxia, peripheral neuropathy, schizophrenia, autism, depression, anxiety, and hallucinations [10,11,12,13,14]. One of the hypothesized links between the gut and the brain (*i.e.*, the brain-gut axis) postulates the existence of an increased intestinal permeability, also referred to as the “leaky gut syndrome” [15]. This in turn could allow gluten peptides (or other wheat-derived proteins) to cross the gut barrier, enter the bloodstream, and cross the blood-brain barrier, either causing neuro-inflammation or affecting the endogenous opiate system and neurotransmission within the nervous system. Food-induced modifications could also target the brain through the microbiota-brain-gut axis where the vagus is also a key element [16]. It should however be stressed that the possible relationship between NCGS and certain neuro-psychiatric disorders such as autism and schizophrenia is still far from clear. Furthermore, the cause/effect relationship between gluten ingestion and neuropsychiatric disorders, in terms of time latency, may be particularly difficult to ascertain.

NCGS should not be an exclusion diagnosis only. There is an increasing need for standardizing the procedure leading to the confirmation of suspected NCGS. Ideally we should have a clear diagnosis before starting treatment, however such certainty is not always possible. In clinical medicine this uncertainty can be resolved by using the treatment as the test that confirms the diagnosis. For example, if we are unsure if a patient’s airway obstruction has a reversible element, a trial of steroids can test this: a sufficient response is then considered evidence of reversibility [17]. Likewise the strategy of “test of treatment” with the GFD can be used to diagnose NCGS.

On 6–7 October 2014, the 3rd International Expert Meeting on Gluten Related Disorders was held in Salerno, Italy, to reach a consensus on how the diagnosis of NCGS should be confirmed. It was acknowledged that in the absence of sensitive and specific biomarkers, a close and standardized monitoring of the patient during elimination and re-introduction of gluten is the most specific diagnostic approach and hence could be used as the diagnostic hallmark of NCGS. In this paper we report the experts’ agreement and recommendations on how the diagnostic protocol should be performed for the confirmation of NCGS.

## 2. NCGS Diagnostic Protocol

The diagnosis of NCGS should be considered in patients with persistent intestinal and/or extra-intestinal complaints showing a normal result of the CD and WA serological markers on a gluten-containing diet, usually reporting worsening of symptoms after eating gluten-rich food. The aim of the confirmation of the diagnosis of NCGS should be twofold: (1) assessing the clinical response to the GFD; (2) measuring the effect of reintroducing gluten after a period of treatment with the GFD. It follows that a full diagnostic evaluation, including Step 1 and 2 (see below), can only be started in the patient who is on a normal, gluten-containing diet. Unfortunately many of these patients are already on the GFD when first seen at the specialty clinic. A simplified/shortened diagnostic procedure, including only Step 2, may be adopted in these patients.

**Figure 1:**
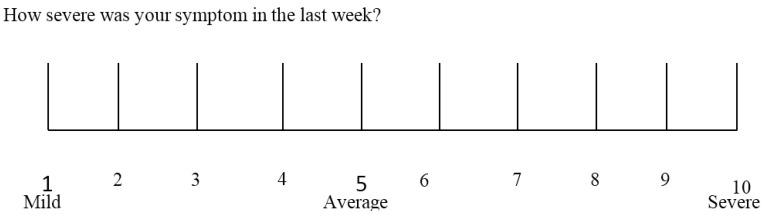
The numerical rating scale (NRS) used for rating the intensity of relevant items.

In both Step 1 and Step 2, the clinical evaluation is performed using a self-administered instrument incorporating a modified version of the Gastrointestinal Symptom Rating Scale (GSRS). The GSRS is a disease-specific instrument, based on reviews of gastrointestinal symptoms and clinical experience, which has been widely used to evaluate common symptoms of gastrointestinal disorders [18]. The instrument presented here includes also items evaluating the extra-intestinal NCGS manifestations. Further items can be included under the box “other” in patients presenting with different symptoms. The patient identifies one to three main symptoms that will be quantitatively assessed using a Numerical Rating Scale (NRS) with a score ranging from 1 (mild) to 10 (severe) (Table 2 and Figure 1).

### 2.1. Step 1: Definition of a Patient Responsive to the GFD (Patient on a Gluten-Containing Diet)

Patients suspected of suffering from a gluten-related disorder should preliminarily undergo a full clinical and laboratory evaluation to exclude CD and WA while still on a gluten-containing diet, according to a previously outlined diagnostic protocol [19].

The following steps establish responsiveness to the GFD:

1. At baseline the patient has to be on a normal gluten containing diet for at least six weeks. The patient is assessed by the Table 2 diagnostic questionnaire at week-2, -1 and 0 to establish baseline symptoms;
2. At time 0 the GFD is started after detailed explanation (preferably by a dietitian);
3. Timeline: at least six weeks of verified GFD. Although the amelioration of symptoms is expected shortly after starting the GFD, a prolonged observation is needed to properly investigate the causal relationship, particularly for fluctuating symptoms (e.g., headache);
4. Data recording: weekly completion of the Table 2 questionnaire from week 0 to 6. The patient will identify one to three main symptoms. The response parameters are those with an initial score of at least 3 on the numerical rating scale (NRS).

The response is assessed for each parameter separately. A symptomatic response is a decrease of at least 30% of the baseline score. Responders are defined as patients who fulfill the response criteria (>30% reduction of one to three main symptoms or at least 1 symptom with no worsening of others) for at least 50% of the observation time (*i.e.*, at least three of six weekly evaluations).

The diagnosis of NCGS is excluded in subjects failing to show symptomatic improvement after six weeks of GFD. GFD-unresponsive patients should be investigated for other possible causes of IBS-like symptoms, e.g., intolerance to FODMAPs or small bowel bacterial overgrowth.

**Table 2:** Questionnaire used for Step 1 evaluation (the same items are evaluated during Step 2).

Intestinal Symptoms	Baseline	1 Week	2 Week	3 Week	4 Week	5 Week	6 Week
Abdominal pain or discomfort							
Heartburn							
Acid regurgitation							
Bloating							
Nausea and vomiting							
Borborygmus							
Abdominal distension							
Eructation							
Increased flatus							
Decreased passage of stools							
Increased passage of stools							
Loose stools							
Hard stools							
Urgent need for defecation							
Feeling of incomplete evacuation							
Extra-intestinal symptoms							
Dermatitis							
Headache							
Foggy mind							
Fatigue							
Numbness of the limbs							
Joint/muscle pains							
Fainting							
Oral/tongue lesions							
Other (specify)							

### 2.2. Step 2: the Gluten Challenge (Patient on the GFD)

Step 2 is required to confirm the diagnosis of NCGS in patients responding to treatment with the GFD. A Double-Blind Placebo-Controlled Challenge with crossover presents a high level of evidence for diagnosing NCGS. Before starting the gluten challenge, the baseline diet needs to be strictly gluten-free to the level of a celiac diet for at least four weeks, *i.e.*, no cross contamination, no gluten traces in the diet. The patient needs to be referred to a dietitian for assessment of the degree of the GFD. Two different types of challenge can be performed depending on the setting: (a) in clinical practice a single blinded procedure could be sufficient; (b) for research purposes, a double blind challenge remains the first choice. Provided there is marked improvement in symptoms with the GFD, the blinded challenges should be undertaken with care. For example, the gluten challenges may need to be repeated to offset the strong *nocebo* effect often seen in these patients.

As far as the daily dose of gluten to be used for the challenge, we suggest an amount of 8 grams, a dose that is both close to the average daily intake of gluten in Western countries (10–15 g) [20] and easy to mix with the vehicle. This dose can be modulated in the research setting. As far as the gluten “vehicle”, gelatin capsules are discouraged. The best-suited vehicle is yet to be developed, for instance it could take form of a muesli bar, bread or muffin, possibly different in children and adults. The vehicle should contain cooked, homogeneously distributed gluten, and should be analyzed in order to know exactly the content of the pro-inflammatory factor ATIs. The gluten preparations should be prepared/tested for ATI bioactivity to contain at least 0.3 g of ATIs/8 g of gluten or gluten should be used with defined ATI content. The vehicle should be FODMAPs free.

The placebo vehicle must be completely gluten-free. Gluten and placebo preparations must be undistinguishable in look, texture and taste, and balanced in fibers, carbohydrate, fat and possibly protein content.

The gluten challenge includes a one-week challenge followed by a one-week washout of strict GFD and by the crossover to the second one-week challenge. The duration of the challenge period may occasionally be longer than a week in patients showing fluctuating symptoms, such as headache or neuro-behavioral problems. A questionnaire with the items shown in Table 2 is self-administered and filled in at baseline, and daily during the first seven-day challenge (or less if symptoms prevent completion of seven days), the washout period, and the second seven-day challenge (or less if symptoms prevent completion of seven days). During the challenge, the patient will identify and report one to three main symptoms, without necessarily filling in the full questionnaire. At least a variation of 30% between the gluten and the placebo challenge should be detected to discriminate a positive from a negative result. The threshold of 30% increment in symptoms is somewhat arbitrary and needs scientific validation. Patients showing a negative gluten challenge should be investigated for other possible causes of IBS-like symptoms, e.g., intolerance to FODMAPs or small bowel bacterial overgrowth. 

**Figure 2:**
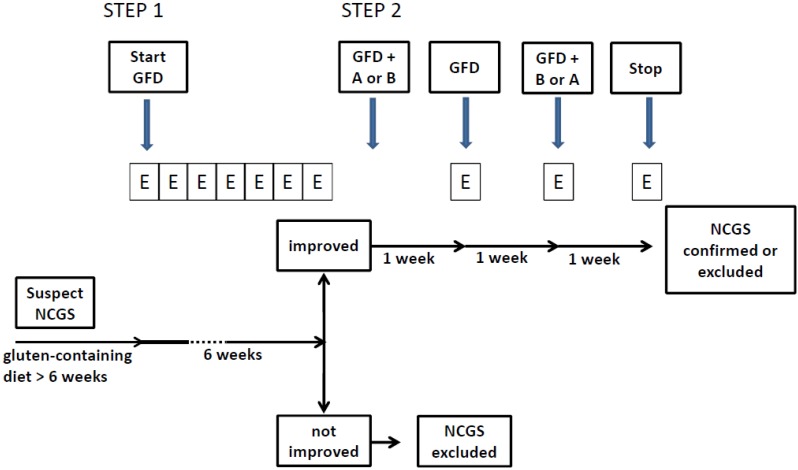
The flow diagram of the NCGS diagnostic process. GFD = gluten-free diet; A = product A (gluten or placebo); B = product B (placebo or gluten); E = evaluation (questionnaire). The evaluation is performed weekly during Step 1 and daily during Step 2.

A detailed flow diagram of the diagnostic process is shown in Figure 2.

### 2.3. Monitoring the Gluten Elimination/Reintroduction Effects by Biomarkers

Ideally the clinical evaluation performed during gluten elimination/reintroduction should include serially repeated specific laboratory tests.

Although the most specific CD serological markers, such as IgA class anti-transglutaminase and anti-endomysial antibodies, are negative in NCGS patients by definition, IgG class antibodies directed against native gliadin (AGA) are found more frequently in these cases (about 50%) than in the general population, when eating a gluten-containing diet. Therefore, the finding of isolated IgG-AGA positivity may be a clue to the diagnosis of NCGS, particularly in subjects with extra-intestinal manifestations. When initially positive, IgG-AGA normalize more quickly in NCGS than CD patients after starting treatment with the GFD [21]. However, it is still unclear whether monitoring the levels of IgG-AGA may be helpful for diagnostic purposes during the rather short period of the gluten elimination/reintroduction challenge.

The hypothesis of a “leaky gut” as a cause of neuropsychiatric disorders found indirect evidence from a study performed by the lactulose/mannitol (L/M) test, a simple clinical investigation exploring the usually divergent trans- and para-cellular permeability of the sugar probes. As a group, autistic children on a regular diet tended to show higher values of the L/M intestinal permeability test when compared with autistic children on a GFD [15]. However, in a subsequent study, a four-week treatment with the GFD did not determine significant changes of the L/M test in a small group of children with autistic spectrum disorder [22]. No L/M test modification has been observed in adult patients affected with typical intestinal manifestations of NCGS [23]. For these reasons, the L/M test cannot be recommended for monitoring the gluten challenge. Recently, Hollon *et al.* investigated the intestinal permeability in human duodenal biopsies mounted in microsnapwells and luminally incubated with either gliadin or media alone. Changes in transepithelial electrical resistance (as an index of intestinal permeability) were monitored over 120 min. Following gliadin exposure, both patients with NCGS and those with active CD demonstrated a greater increase in intestinal permeability than celiacs in disease remission [24]. The clinical significance of these findings remains to be elucidated.

The research on biological marker/s of NCGS is currently very active. Preliminary data observed in intestinal biopsies of NCGS patients showed an increase of intraepithelial lymphocytes (Marsh I) as well as the presence of markers associated with innate, rather than adaptive, immunity [2,23]. Recently, in an intestinal biopsy-based study, NCGS patients showed increased mucosal IFN-γ mRNA after a three-day gluten challenge [25]. Taken together these results suggest the presence of a mucosal immune activation in patients with NCGS. Therefore, the precise mechanisms underlying the induction of the immune response and the identification of reliable biomarkers for the diagnosis of NCGS are relevant issues that should be resolved. A search on www.clinicaltrials.gov performed on 2 January 2015 identified seven studies currently in progress to evaluate serological and mucosal indexes that could eventually find an application for diagnosing NCGS in clinical practice.

## 3. Conclusions and Future Perspectives

NCGS is a recently “re-discovered” clinical entity distinct from CD for which we have very little certainty and many knowledge “black holes”. NCGS was first described in the early 1980s [26], but over the past decade the number of patients diagnosed with NCGS and publications on this topic have increased greatly. However, it is still not clear how to diagnose and manage this condition, and the pathophysiological mechanisms are unclear. Therefore, in terms of knowledge, we are with NCGS now where we were with CD 40 years ago. Since we still do not have validated biomarker(s) for the diagnosis of NCGS, the diagnostic protocol remains cumbersome and not apt for large epidemiological studies aimed at establishing the prevalence of this condition. However specific diagnostic criteria for NCGS are necessary for optimizing clinical care, avoiding self-diagnosis and advancing the science of NCGS. The guidelines provided in this paper will (a) help the clinician to reach a firm and positive diagnosis of NCGS and (b) facilitate the comparisons of different studies, if adopted internationally. A more practical approach will only be possible with the development of biomarkers or other clinical predictors.

The identification and validation of biomarker(s) will be instrumental to gain insights in NCGS pathogenesis, to establish the trigger(s) of this condition, and ultimately to establish the magnitude of this clinical condition. We will not be able to have a full understanding of NCGS until better diagnostic tools will become available and we have more information on NCGS pathogenesis, following the same path we followed during the last four decades of CD research.
